# Engineered hyalurosomal gel co-delivering citicoline sodium and nifedipine for potential glaucoma management: formulation and pharmacodynamic evaluation

**DOI:** 10.3389/fphar.2026.1977895

**Published:** 2026-10-23

**Authors:** Doaa Hegazy, Mennatullah M. Ibrahim, Riham A. El-Shiekh, Osama Saher, Nouran A. Ali

**Affiliations:** 1 Department of Pharmaceutics and Industrial Pharmacy, Faculty of Pharmacy, Cairo University, Cairo, Egypt; 2 Department of Pharmacognosy, Faculty of Pharmacy, Cairo University, Cairo, Egypt; 3 Department of Laboratory Medicine, Karolinska Institute, Stockholm, Sweden; 4 Department of Cellular Therapy and Allogeneic Stem Cell Transplantation (CAST), Karolinska University Hospital Huddinge and Karolinska Comprehensive Cancer Center, Stockholm, Sweden

**Keywords:** antioxidant activity, calcium channel blocker, combined therapy, free radical scavenging, glaucoma, neuroprotection

## Abstract

**Introduction:**

Glaucoma is a neurodegenerative ocular disorder that requires multi-targeted therapeutic strategies to counteract intraocular pressure (IOP) elevation and oxidative stress–mediated retinal ganglion cells (RGCs) loss. This study developed an innovative hyalurosomal hydrogel platform co-delivering Nifedipine (NFD, a calcium channel blocker that promotes ocular vasodilation and IOP reduction) and Citicoline sodium (CTC, a potent neuroprotective and antioxidant agent) as a potential topical therapeutic approach for glaucoma.

**Methods:**

NFD-loaded hyalurosomes (NFD-HAs) were prepared using thin-film hydration and statistically optimized via a D-Optimal design. The optimized formulation (NFD-OF) was probe-sonicated, combined with CTC, and incorporated into an HPMC hydrogel.

**Results and discussion:**

Sonicated NFD-OF displayed nanosized spherical vesicles (198.6 ± 23.1 nm), high entrapment efficiency (70.7 ± 1.8%), and stable zeta potential (–39.1 ± 1.3 mV). Transmission electron microscopy (TEM) analysis confirmed the distinct spherical morphology, non-aggregated state, and structural integrity of NFD-OF. The final CTC/NFD-OF gel exhibited pseudoplastic shear-thinning flow, an optimal ocular pH (6.9 ± 0.02), and sustained release over 24 h (65.5% for NFD and 99.5% for CTC). The dual gel demonstrated superior free radical scavenging capability (IC_50_ = 16.44±0.7 μg/mL) compared to free NFD (219.43 μg/mL) and CTC (234.62 μg/mL). Confocal microscopy confirmed a 2.2-fold enhancement in transcorneal penetration depth (110 μm) compared with the control solution (50 μm). *In vivo* pharmacodynamic evaluation revealed a significantly higher maximum IOP decrease (51.85 ± 8.2%), extended mean residence time (8.9 ± 2.7 h), and increased relative exposure (AUC_0_–∞ = 494.1 ± 191.9) compared to commercial Xola™ eye drops (36.4 ± 5.9% max IOP decrease, AUC_0_–∞ = 182.8 ± 33.7). Terminal gamma sterilization preserved physicochemical characteristics and release profiles. Draize and histopathological assays confirmed excellent ocular tolerability. Overall, the CTC/NFD-OF gel demonstrated promising ocular hypotensive activity and safety, supporting its potential as a multifunctional topical therapeutic approach for glaucoma.

## Graphical Abstract

**Figure:**
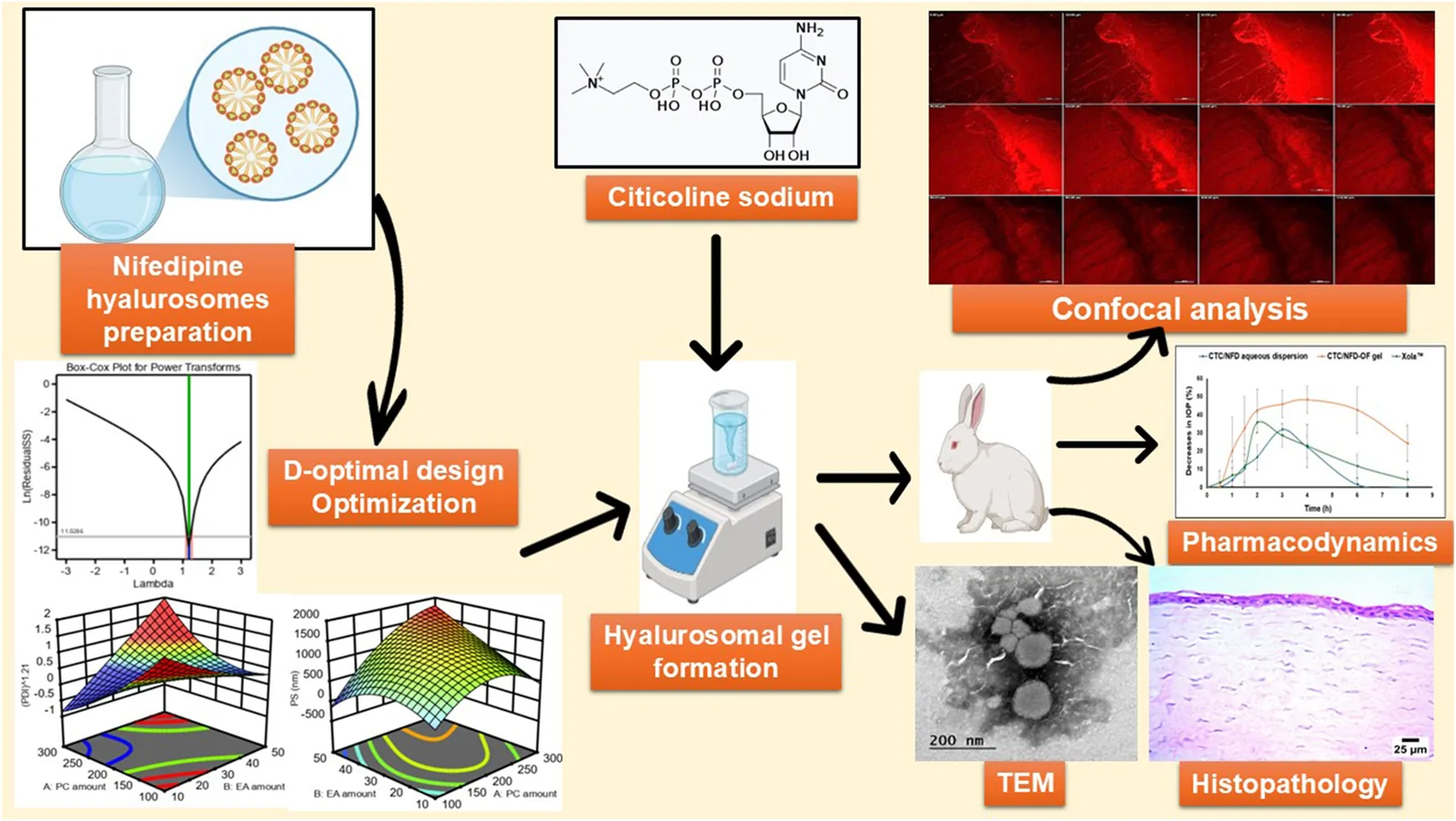

## 1 Introduction

The eye is a small, complex organ with distinct anatomical, physiological, and biochemical characteristics (Tawfik et al., 2026). Its unique structure and protective barriers pose significant challenges to topical drug delivery (Salama et al., 2026a). While systemic administration of ocular drugs offers the convenience of easy delivery, it is limited by low bioavailability and the risk of systemic side effects. On the other hand, topical application via traditional dosage forms such as solutions and suspensions faces substantial challenges, including nasolacrimal drainage, tear turnover, and drug loss on the eyelids (Tawfik et al., 2026; Tawfik et al., 2025; El-Feky et al., 2021). Accordingly, there is an earnest need to develop more topical ophthalmic preparations with effective drug delivery, while maintaining ocular safety and patient compliance (Salama et al., 2026b). Recent advancements in nanocarrier-mediated drug delivery have demonstrated significant potential in overcoming these biological barriers and providing enhanced cellular protection (Li et al., 2019). Furthermore, the physicochemical properties of these nanocarriers, particularly their surface charge, play a crucial role in determining their interaction with ocular membranes and modulating their cellular uptake efficacy (Zhang et al., 2026).

Glaucoma is an eye disorder characterized by elevated intraocular pressure (IOP) and progressive damage to the optic nerve, principally affecting middle-aged and older individuals; therefore, glaucoma, as a devastating condition, requires cautiously controlled treatment (Fouda et al., 2018). Pathological manifestations of the disease can be described as impairments in the regulation of aqueous humour secretion or outflow, leading to aqueous humour accumulation and an increase in IOP (Tawfik et al., 2025; Fouda et al., 2018). Miscellaneous classes of drugs have been used for glaucoma treatment, including beta-adrenergic blockers (Tejwani et al., 2020), carbonic anhydrase inhibitors (Fouda et al., 2018), prostaglandin analogues (Wasyluk et al., 2025; Baudouin et al., 2025), and α_2_-adrenergic agonists (Emad Eldeeb et al., 2019). Nowadays, drug repurposing has gained considerable attention, offering more therapeutic alternatives to combat such dreadful diseases as glaucoma. One class that has recently emerged as a potential alternative treatment for glaucoma is the calcium channel blockers class. While calcium channel blockers have traditionally been used to treat coronary disease, recent studies suggest that they may also help improve visual fields in cases of low-tension glaucoma or suspected vasospasm, potentially by enhancing blood flow to the eye (El-Feky et al., 2021; Fang et al., 2015).

Nifedipine (NFD), a dihydropyridine calcium channel blocker, has been used to manage hypertension and angina pectoris. Recent findings suggest that NFD may also be effective in treating elevated IOP, as it counteracts the vasoconstrictive action of endothelin-1 (ET-1), promoting vasodilation in the ocular vascular smooth muscles, and thereby reducing IOP (El-Feky et al., 2021; Fang et al., 2015). Therefore, applying NFD locally to the eye, entrapped into hyaluronic acid (HA)-modulated nanosystems (hyalurosomes) loaded in a hydrogel, may offer a potential candidate for glaucoma treatment, providing a sustained effect with diminished systemic side effects such as lowered blood pressure.

Being a progressive optic neuropathy, glaucoma is characterized by retinal ganglion cells (RGCs) degeneration and optic nerve damage, in which oxidative stress plays a key pathogenic role (Chan et al., 2025). Indeed, suppressing oxidative stress has been validated as a critical mechanism for neuroprotection in various ischemic and neurodegenerative experimental models. Furthermore, comparative preclinical assessments emphasize the necessity of integrating multi-targeted neuroprotective treatments to effectively mitigate neuronal injury (Zhang et al., 2023a; Zhang et al., 2023b).

Citicoline (cytidine-5′-diphosphocholine) (CTC) has emerged as a promising neuroprotective agent with indirect antioxidant properties in glaucoma (Roberti et al., 2014). CTC exerts its antioxidant effects primarily by stabilizing and repairing neuronal cell membranes through enhanced phosphatidylcholine synthesis, thereby reducing membrane lipid peroxidation and preserving membrane integrity under oxidative conditions (Abdel-Aziz et al., 2025). At the molecular level, CTC reduces oxidative stress–induced apoptosis by modulating mitochondrial apoptotic pathways, and indirectly enhancing endogenous antioxidant defense systems, such as glutathione-mediated redox balance, further limiting ROS-induced cellular injury and enhancing RGCs survival (Abdel-Aziz et al., 2025; Gandolfi et al., 2020). The aforementioned CTC’s inherent properties make it particularly suitable for RGCs protection (Kitamura et al., 2019). Interestingly, NFD was reported to have possible antioxidant, neuroprotective and anti-inflammatory activities (Yao et al., 2020; Abd El Aziz et al., 2021). Therefore, motivated by all these considerations, CTC presents an appealing opportunity for a combined therapy with NFD, offering integrated neuroprotection and IOP-lowering benefits.

Hyaluronic acid gel-filled core liposomes, known as Hyalurosomes, are innovative liposomal systems composed of phospholipid vesicles encapsulating a gel core (Kawar and Abdelkader, 2019). This architecture combines the benefits of liposomes and hyaluronic acid (HA) in a single drug-delivery platform. HA is a fundamental part of the body’s extracellular matrix, which has been recognized as a non-toxic, biodegradable polymer with considerable biocompatibility (Burdick et al., 2011). It is also known for its non-irritating properties and for exhibiting high water-binding capability, along with viscosity and pseudoplastic behavior. Additionally, HA can function as a mucoadhesive polymer, enhancing the ocular residence time (Casey-Po et al., 2022). HA-based hydrogels are commonly used to treat eye dryness. Thanks to its viscoelastic properties, it has been used as a suitable ingredient in artificial tears (commercially available in different concentrations up to 0.4%) (Hynnekleiv et al., 2022). Furthermore, HA has been reported in ophthalmology, incorporated into liposomes or as a coating material (Moustafa et al., 2017), highlighting its therapeutic benefits; however, the integration of HA into a nano-vesicular structure for ocular delivery of NFD has not been thoroughly explored.

The goal of this study was to develop an innovative ocular triple platform, named CTC/NFD-OF gel. A comprehensive sequential characterization and *in-vitro* optimization of the prepared NFD-loaded hyalurosomes (NFD-HAs) were conducted, followed by the addition of CTC, and further investigations of the final formula. Moreover, the ability of the innovative CTC/NFD-OF gel to enhance CTC/NFD corneal delivery and localization in the aqueous humour was assessed by the *in-vivo* corneal reuptake test and pharmacodynamic studies in rabbits.

The novelty of our work lies in the unique combination of two therapeutically complementary agents that target different pathological aspects of glaucoma in a single platform, which navigates the cooperation between (1) ocular vasodilation, potential neuroprotective and antioxidant activities of NFD, (2) neuroprotective, antioxidant, and neurorestorative activities of CTC, (3) hydrating, soothing, anti-inflammatory, and mucoadhesive properties of HA and, (4) the intrinsic well-known features of hyalurosomes, including enhanced structural stability of the nanosystem due to the gel core, improved penetration, controlled release of loaded drugs, and extended therapeutic action (Ibrahim et al., 2024)to act harmoniously, paving the way towards a combined therapy for better glaucoma control via a patient-friendly formula that maintains patient convenience and compliance.

The present study, therefore, focused on the development and optimization of a dual-loaded hyalurosomal gel platform (CTC/NFD-OF gel) as a potential topical approach for glaucoma management. NFD-loaded hyalurosomes were initially prepared via thin-film hydration and statistically optimized using a D-optimal design to evaluate principal quality attributes, including particle size (PS), polydispersity index (PDI), entrapment efficiency (EE), and zeta potential (ZP). The optimized nanovesicles were probe-sonicated, combined with CTC, and successfully incorporated into an HPMC hydrogel matrix. The final platform underwent comprehensive characterization, including TEM morphological assessment, rheological profiling, release kinetics, terminal gamma sterilization stability, *ex-vivo* transcorneal permeation depth, and *in-vivo* pharmacodynamic and ocular tolerability evaluations in a rabbit model. By combining the IOP-lowering and vasodilatory effects of NFD with the neuroprotective and antioxidant capacity of CTC within a mucoadhesive, gel-filled vesicular platform, this approach was designed to overcome the biological barriers of conventional eye drops.

Consequently, this study successfully achieved a highly stable, dual-loaded hyalurosomal gel that demonstrated superior free-radical scavenging capacity and significantly enhanced transcorneal penetration compared to free drug solutions. Furthermore, *in-vivo* evaluations confirmed excellent ocular tolerability and a profound, sustained reduction in intraocular pressure (IOP) in ocular normotensive rabbit models, proving the exceptional potential of the CTC/NFD-OF gel as an effective, patient-compliant platform for advanced glaucoma management.

## 2 Materials and methodology

### 2.1 Materials

Nifedipine (NFD) was obtained from Pharco Pharmaceutical Co., Alexandria, Egypt. Citicoline sodium (CTC) was supplied by Global Napi Pharmaceuticals, 6th of October City, Egypt. Sodium hyaluronate (HA), 95% (molecular weight 1.5 to 2.2 million Da) was purchased from Acros Organics Co., Geel, Belgium. Cellulose dialysis membrane average width 25 mm, (typical molecular weight cut-off = 14,000 Da), Phospholipid from egg yolk (∼60% L-α-phosphatidylcholine (PC)), Sodium Cholate (CO), Sodium deoxycholate (DEO), 2,2-Diphenyl-1-picrylhydrazyl (DPPH), and Hydroxy propyl methyl cellulose (90,000 cp) (HPMC) was purchased from Sigma Aldrich Co., St. Louis, MO, USA. Methyl alcohol, Sodium Chloride (NaCl), Disodium Hydrogen Phosphate (Na_2_HPO_4_), Potassium Dihydrogen Phosphate (KH_2_PO_4_) were purchased from El-Nasr Pharmaceutical Co., Giza, Egypt.

### 2.2 Methodology

#### 2.2.1 Preparation of NFD-HAs using D-optimal design

The D-optimal design was selected to statistically examine the effect of chosen independent variables, based on the results of a screening study, namely, A: L-α-phosphatidylcholine (PC) amount (mg), B: Edge activator (EA) amount (mg) and C: Edge activator (EA) type on scrutinized dependent responses which were set to be Y1: EE (%), Y2: PS (nm), and Y3: PDI, as listed in Table 1. The Design Expert® software (Version 13, Stat-Ease Inc., MN, USA) (DX13) was utilized to generate 17 formulae. Their detailed composition is shown in Table 2. Subsequently, the elucidated polynomial equations were modulated by omitting non-significant model terms for better assessment of the relationship between the independent variables investigated and the dependent responses. Also, the suggested restraints were defined on the desired responses to permit the software, through numerical desirability algorithms, to derive an optimized NFD-HAs formula (NFD-OF).

**TABLE 1:** D-optimal design, independent variables and constraints for optimization of nifedipine loaded hyalurosomes.

Factors (independent variable)	Levels	
	Low (−1)	High (+1)
A: Phosphatidylcholine amount (mg)	100	300
B: Edge activator amount (mg)	10	50
C: Edge activator type	Sodium cholate	Sodium deoxycholate
Responses (dependent variables)	Desirability constrains	
Y1: Entrapment efficiency (%)	Maximize	
Y2: Particle size (nm)	Minimize	
Y3: Polydispersity index	Minimize	

**TABLE 2:** Composition of different NFD-HAs according to D-optimal design and their characterization results (Mean ± SD).

NFD-HAs	PC amount (mg)	EA amount (mg)	EA type	EE (%)	PS (nm)	PDI^1.21*	ZP (mV)
H1	194	10	CO	30.9 ± 2.3	978.4 ± 11.10	0.37 ± 0.4	−38.4 ± 1.1
H2	300	28.8	CO	77.4 ± 0.1	1,066.2 ± 23.6	0.52 ± 0.3	−43.6 ± 1.1
H3	300	28.8	CO	77.4 ± 3.0	1,066.2 ± 12.6	0.52 ± 0.3	−43.6 ± 1.1
H4	200	30	CO	76.9 ± 3.6	1,376.1 ± 53.1	0.34 ± 0.2	−23.9 ± 2.2
H5	100	31.2	CO	25.4 ± 2.1	1,219.9 ± 68.0	0.87 ± 0.3	−44.2 ± 0.1
H6	205	50	CO	41.8 ± 3.5	1,261.7 ± 50.0	0.62 ± 0.3	−37.9 ± 1.6
H7	110	50	CO	28.4 ± 2.1	160.8 ± 0.600	0.37 ± 0.2	−28.0 ± 4.1
H8	100	10	DEO	20.8 ± 1.2	1,464.6 ± 85.7	0.49 ± 0.2	−41.8 ± 2.0
H9	200	10	DEO	84.0 ± 1.3	828.10 ± 22.9	0.52 ± 0.2	−40.4 ± 0.3
H10	300	10	DEO	77.3 ± 2.7	778.9 ± 16.10	0.78 ± 0.3	−40.0 ± 2.2
H11	100	30	DEO	19.5 ± 1.3	1743.9 ± 84.1	0.56 ± 0.2	−36.4 ± 4.0
H12	200	30	DEO	36.7 ± 2.6	993.10 ± 34.0	0.27 ± 0.2	−44.6 ± 1.7
H13	300	30	DEO	83.5 ± 1.5	1,026.7 ± 30.2	0.63 ± 0.2	−42.7 ± 0.7
H14	100	50	DEO	15.9 ± 0.6	940.70 ± 28.0	0.92 ± 0.1	−41.7 ± 8.9
H15	100	50	DEO	15.9 ± 0.6	940.90 ± 28.0	0.92 ± 0.1	−36.5 ± 8.9
H16	300	50	DEO	80.7 ± 6.0	1,322.6 ± 60.3	0.52 ± 0.3	−45.6 ± 1.8
H17	300	50	DEO	80.7 ± 6.0	1,322.6 ± 69.3	0.52 ± 0.3	−43.9 ± 1.8

* All NFD-HAs, contain 10 mg of nifedipine and 0.1% (w/v) of Hyaluronic acid. PDI, values were raised to the power (1.21) as recommended by Design expert (Box-Cox transformation), PC, phosphatidylcholine; EA= edge activator; EE, entrapment efficiency; PS, particle size; ZP, zeta potential, and PDI, polydispersity index.

#### 2.2.2 Method of preparation of NFD-HAs

NFD-HAs were fabricated by implementing the thin-film hydration technique (Bangham’s method) (Elsayed et al., 2025; Ali et al., 2026). In brief, 7 mL of chloroform: methanol in a ratio (5:2) was placed in a round-bottom flask to dissolve accurate weights of NFD (10 mg), PC in different amounts ranging from 100 mg to 300 mg, together with an EA (either sodium cholate (CO) or sodium deoxycholate (DEO)) in different amounts ranging from 10 mg to 50 mg. The flask was attached to a rotary evaporator (Heidolph Instruments GmbH & Co., Schwabach, Germany), which was conditioned at 60 °C, and allowed to rotate at 150 rpm under vacuum for 15 min till the complete evaporation of the organic solvents. After that, a homogenous thin film was developed by the end of the evaporation stage. After releasing the vacuum, 10 mL of double-distilled water containing 10 mg HA was added, and the flask resumed rotation at the same speed and temperature for another 25 min till the formation of a yellow milky-like dispersion of NFD-HAs. The prepared NFD-HAs were left in the refrigerator at 4 °C for 24 h to allow the maturation of the formed hyalurosomes overnight before characterization.

#### 2.2.3 Determination of NFD entrapment efficiency percentage (EE)

To quantify EE of NFD-HAs, 1 mL of each NFD-HAs formula was centrifuged for 1 h at 20,000 rpm and 4 °C using a cooling ultra-centrifuge (Beckman, Fullerton, Canada), then the supernatant was dissolved in methanol, and the concentration of the unentrapped drug was measured spectrophotometrically (UV/VIS spectrophotometer, Model 1800, Shimadzu, Kyoto, Japan) at the determined λ_max_ of NFD in methanol (351.6 nm) (Wasan et al., 2019) after performing necessary dilutions. The UV method was validated by accuracy and precision, and the constructed calibration curve for NFD in methanol possessed sufficient linearity with a coefficient of determination (R^2^) of 0.999. The EE was calculated using Equation 1 (Youssef et al., 2026; Badr-Eldin et al., 2026):

$$\text{EE}=\frac{Total\;NFD-unentrapped\;NFD}{Total\;amount\;of\;NFD}\times 100$$ (1)

The measurements were done in triplicate, and the results were recorded as (Mean ± SD).

#### 2.2.4 Determination of particle size (PS), zeta potential (ZP), and polydispersity index (PDI)

Quantitative determination of PS, ZP and PDI of the prepared NFD-HAs was carried out using the technique of dynamic light scattering by Malvern ZS (ZEN3600, Malvern Instrument Ltd., Worcestershire, UK) (Fahmy et al., 2026). Each prepared NFD-HAs formula was measured after being properly diluted with double-distilled water in a ratio of 1:100 to achieve a suitable intensity of scattering. ZP measuring was also done by utilizing the same equipment to detect the particles’ electrophoretic movement in an applied electric field (Fahmy et al., 2026; Omar et al., 2026). Each sample was measured three times, and the results were represented as the mean value ± SD.

#### 2.2.5 Statistical optimization

Statistical optimization was applied through a numerical desirability function generated by DX13. The optimization was delineated by the applied constraints on the targeted dependent responses, as mentioned in Table 1. The optimization algorithm was grounded on maximizing EE, while keeping PS and PDI minimum. The suggested NFD-OF, based on the selection standards input to DX13, was prepared and re-evaluated with respect to its EE, PS, PDI, and ZP. To impartially evaluate the validity of the chosen model, an ANOVA test was performed to objectively ratify the significance of the difference that may exist. The desirability function applied is represented in Equation 2 (Hegazy et al., 2025):

$$D={\left(d_{1}.d_{2}\ldots d_{m}\right)}^{\frac{1}{m}}$$ (2)

Where D is the total desirability calculated by DX13, ranging from 0 to 1. d_m_ is the desirability of each dependent response, ranging from 0 to 1. m is the total number of dependent responses to be processed in the optimization.

#### 2.2.6 Preparation of CTC-loaded NFD-OF gel (CTC/NFD-OF gel)

NFD-OF was fabricated using the same thin film hydration technique mentioned earlier. Then it was sonicated for 40 s for extra size reduction to encourage better corneal penetration in further *in-vivo* studies. After that, CTC (2% w/v) was dissolved in the aqueous phase of NFD-OF under stirring for 10 min (Carnevale et al., 2019). Finally, HPMC was added to the CTC/NFD-OF under stirring for 15 min to prepare the final gel (CTC/NFD-OF gel, 0.75% w/v), which was subsequently evaluated for *in-vitro* release, microscopic examination, pH, refractive index, surface tension, and rheological properties.

#### 2.2.7 Evaluation of the *in-vitro* drug release from CTC/NFD-OF and its corresponding gel

The release of NFD and CTC from the CTC/NFD-OF, CTC/NFD-OF gel and free CTC/NFD in distilled water was carried out in a horizontal shaking water bath (GFL, Gesellschatt Laboratories, Berlin, Germany). One mL of CTC/NFD-OF, CTC/NFD-OF gel and each drug solution was placed in a dialysis bag (25 mm width and 12,000–14,000 molecular weight cut-off) inside tightly stoppered, amber-colored bottles. The release medium was 50 mL of 25% methanol in phosphate buffer saline (pH = 7.4), to achieve sink conditions for NFD, at 37 °C ± 0.5 °C under an agitation speed of 50 rpm (Hegazy et al., 2025). Aliquots of 3 mL were withdrawn at time intervals up to 24 h. These samples were subsequently replaced by 3 mL of fresh release medium each time. Samples from the release medium were analyzed spectrophotometrically (UV/VIS spectrophotometer, Model 1800, Shimadzu, Kyoto, Japan) by applying the first derivative analytical method to bypass the overlapping of NFD and CTC spectra. First, the absorbance of samples at λ_max_ = 340 nm (zero crossing of CTC) was measured to quantify NFD, then the first derivative transformation was applied, and the absorbance was detected at λ_max_ = 287.5 nm (zero crossing of NFD) for the quantitative measurement of CTC (Eldeeb et al., 2022). Release profiles were drawn by plotting the cumulative percent of NFD and CTC released at each time point (Q_t_) against time (h). Q_t_ was calculated according to Equation 3 (Abdelhakeem et al., 2026):

$$Q_{t}=\frac{Cn\;x\;Vr+\sum_{i=1}^{n-1}Ci\;x\;Vs}{intial\;drug\;amount}\;x\;100$$ (3)

where Q_t_ is the current cumulative percentage of NFD or CTC released, C_n_ is assigned to the receptor medium concentration at the n^th^ sample, Vr is the total volume of release medium, Vs is the volume of each sample removed for analysis, and $\sum_{i=1}^{n-1}Ci$ denotes the sum of the previously determined concentrations (Abdel Hafeez Ismail et al., 2026).

Kinetic analysis of NFD and CTC release from CTC/NFD-OF gel was evaluated via zero-order (Equation 4) and first-order kinetics (Equation 5) models, while the mechanism of release was evaluated using the Korsmeyer-Peppas model (Equation 6). Microsoft Excel® (Microsoft Office 365, One Microsoft Way, Redmond, Washington, USA) was used to process mathematical calculations and determine the coefficient of determination (R^2^) of each model, where the highest R^2^ denotes the best-fit model. The following equations were used to describe each model (Jain and Jain, 2016):

Zero order:

$$Q_{t}=\;Q_{0}+k_{0}t$$ (4)

First-order kinetics:

$$\log\;\left(100-Q_{t}\right)=\log Q_{0\;}-\frac{kt}{2.303}$$ (5)

Korsmeyer-Peppas:

$$\log Q_{t}=logk+n\log t$$ (6)

where $Q_{t}$ is the cumulative amount of drug released at the time t , $Q_{0}$ is the initial amount of drug, $k_{0}$ is the zero-order release constant, k is the first-order release constant, and n , for the Korsmeyer-Peppas model, is the release exponent indicating the mechanism of drug release.

#### 2.2.8 Transmission electron microscopy (TEM)

The morphology of the CTC/NFD-OF and CTC/NFD-OF gel after terminal sterilization, was examined using transmission electron microscopy (TEM) (Joel JEM 1230, Tokyo, Japan) to confirm the integrity of the formed hyalurosomes, the spherical vesicular structure, and the presence of a gel-like core. A drop of each undiluted sample was stained with 1% (w/v) of phosphotungistic acid, air-dried at room temperature, positioned on a specific copper grid coated with carbon, and then observed at an accelerating voltage of 80 kV (Nemr et al., 2025; Ahmed et al., 2025a).

#### 2.2.9 Fourier transform infrared (FTIR) spectroscopy

FTIR analysis aims to identify any probable physicochemical interactions among the CTC/NFD-OF gel constituents and to verify the encapsulation of NFD into NFD-HAs (Ahmed et al., 2025a; Eladawy et al., 2021). Preceding FTIR analysis, CTC/NFD-OF was kept at −18 °C for 24 h, followed by lyophilization using a freeze dryer (Novalyphe-NL 500; Savant Instruments Inc., Holbrook, NY, USA) at −45 °C under a vacuum pressure of 7 × 10^−2^ mbar till complete dryness (Ahmed et al., 2026). FTIR spectra of NFD, CTC, PC, HA, CO, HPMC, and the freeze-dried CTC/NFD-OF were recorded over the spectral range of 4,000–400 cm^-1^ using an IR spectrophotometer (IRAffinity-1; Shimadzu, Kyoto, Japan) employing the potassium bromide (KBr) pellet method (Abdelhakeem et al., 2026). For sample preparation, each component was finely ground and thoroughly mixed with KBr in a mortar for 3–5 min to obtain a final homogenous blend. The mixtures were then compressed into transparent pellets under a load of 5 tons for 5 min using a hydraulic press. The prepared discs were positioned in the optical path of the instrument, and the obtained spectra were analyzed.

#### 2.2.10 Effect of terminal sterilization by gamma radiation

Terminal sterilization of the prepared CTC/NFD-OF gel was performed using gamma irradiation from a ^60^Co source at the National Center for Radiation Research and Technology (Cairo, Egypt). The formula was exposed to a radiation dose of 25 kGy at a dose rate of 1.19 kGy/h (Fouda et al., 2018; Sayed et al., 2020). To prevent thermal effects such as melting or aggregation that could result from temperature elevation during irradiation, CTC/NFD-OF gel was sealed in a polyurethane container, surrounded by dry ice throughout the sterilization process. Following gamma irradiation, the sterilized CTC/NFD-OF gel was re-evaluated for EE, PS, ZP, PDI, release profiles and TEM examination to assess any changes induced by the sterilization procedure (Ali et al., 2024). Similarity factor (f_2_) was calculated to evaluate differences in the release profiles of NFD and CTC before and after the sterilization process. The similarity equation Equation 7 utilized was as follows (Ahmed et al., 2025b; Sayyed et al., 2026):

$$f_{2}=50.\;\mathrm{Log}\;\left\{{\left[1+\left(1/n\right)\sum_{t=0}^{n}{\left(Rt-Tt\right)}^{2}\right]}^{-0.5}\right\}.100$$ (7)

For each drug (CTC or NFD), an individual f_2_ was calculated, where R_t_ is the % released of the drug from CTC/NFD-OF gel before sterilization, while T_t_ is the % released after sterilization (Fouda et al., 2018). n indicates the number of samples. Similarity factor (f_2_) should exceed 50 to confirm a similarity between the release profiles (Ahmed et al., 2025a).

#### 2.2.11 pH measurement, refractive index and surface tension of CTC/NFD-OF gel

Measurement of pH is an essential attribute for assessing the ocular safety and tolerability of ophthalmic formulae (Račić et al., 2025). Ophthalmic products are generally required to have a pH between 3.5 and 8.5 to avoid eye irritation and minimize reflex tearing, which may otherwise lead to rapid elimination of the administered preparation (Mohapatra et al., 2025). The pH of CTC/NFD-OF gel was determined in triplicate using a Jenway pH meter (Model 3,505, Bibby Scientific Ltd., UK). One g of the gel was dispersed into 9 g of double distilled water, and the mean ± SD was reported (Ashraf Nemr et al., 2026).

Two other important features of the ophthalmic preparation that must be evaluated are the refractive index and surface tension to avoid blurred vision and promote corneal penetration, respectively (Ammar et al., 2009; Dave and Paliwal, 2014). For the determination of the surface tension of CTC/NFD-OF gel, a Du Nouy ring force tensiometer (Model K-6, Krüss GmbH, Hamburg, Germany) was used, with water as the reference (Lee et al., 2012). 50 mL of either distilled water or CTC/NFD-OF gel was individually placed in a beaker at room temperature. The tensiometer ring was immersed in each sample at a constant distance from the surface (4.5 mm), and the force required to withdraw the ring from the sample, reflecting the surface tension, was recorded.

The light refractive index of CTC/NFD-OF gel was also determined using a Hilger and Watts refractometer (Model-46.17/63707, Hilger and Watts Ltd., London, UK) to check whether the CTC/NFD-OF gel adversely affects vision.

#### 2.2.12 Rheological properties of CTC/NFD-OF gel

The viscosity of CTC/NFD-OF gel was evaluated using a Brookfield viscometer (Model HBDV-1 + CP, Spindle CPE-41, Middleboro, MA, USA), with the rotational speed gradually increasing from 0.5 to 100 rpm. Approximately 1 g of CTC/NFD-OF gel was placed on the viscometer plate for evaluation. Measurements were conducted at incremental speeds between 0.5 and 100 rpm, allowing a 10 s interval between successive rotations. Data were considered valid and recorded only when the torque values fell within the acceptable range of 10%–100% (Elsayed et al., 2025). Rheological profiles (rheograms) of CTC/NFD-OF gel were constructed, where shear rate and shear stress were depicted on the Y- and X-axes, respectively. As well, viscosity was plotted as a function of shear rate. To describe the flow behavior of CTC/NFD-OF gel, multiple rheological models were implemented. The viscometer provided shear stress and viscosity values at varying shear rates, which were subsequently fitted to the power-law model (Elsayed et al., 2025), mentioned below in Equation 8, to elucidate the rheological features of CTC/NFD-OF gel.

$$\tau =K\gamma^{n}$$ (8)

Where τ is the shear stress, γ is the rate of shear, K is the consistency index, and n is the flow index (dimensionless). For a shear-thinning fluid, n lies between zero and one, while it approaches one in the case of a Newtonian system and exceeds one in a dilatant system (Gad et al., 2008). Several models (Bingham’s, Casson’s and Carreau’s) were used to describe the non-Newtonian system (Fahmy et al., 2018). Comparison of their regression coefficient could verify the type of the non-Newtonian system.

#### 2.2.13 *In-vitro* antioxidation: scavenging DPPH free radical

Antioxidant activities of the CTC, NFD, and CTC/NFD-OF gel were assessed using 2,2-Diphenyl-1-picrylhydrazyl (DPPH) antioxidant capacities (Liu et al., 2023). The procedure adopted was previously described by Hashem et al. (Hashem et al., 2026). First, a blank solution consisting of 20 μL of methanol and 200 μL of DPPH solution was prepared. Similarly, NFD and CTC solutions in methanol were also prepared individually at serial concentrations of 0.78125–50 μg/mL, and 20 μL of each was mixed with 200 μL of the DPPH solution. The same procedure was followed in the assessment of the antioxidant activity of the CTC/NFD-OF gel and the blank hyalurosomal gel, as 20 μL of each formula was blended individually with the same volume of the DPPH solution. Following thorough mixing and gentle agitation, the samples were incubated for 1 h in ambient conditions with protection from light exposure. Absorbance readings were subsequently recorded at a wavelength of 490 nm. The half-maximal inhibitory concentration (IC_50_) was determined using linear regression analysis. Data processing and statistical calculations were carried out using Microsoft Excel 2010. Percentage DPPH radical scavenging activity was calculated by the following Equation 9 (Hashem et al., 2026):

$$\%\text{ DPPH radical scavenging activity}=\frac{A0-\left(A1-A2\right)}{A0}\;X100$$ (9)

Where A0 is the absorbance of the control, A1 is the absorbance of the CTC/NFD-OF gel, and A2 is the absorbance of the blank hyalurosomal gel. Then % of inhibition was plotted against concentration, from which IC_50_ was calculated. The experiment was repeated three times at each concentration, and the results were expressed as mean ± SD.

#### 2.2.14 *In-vivo* evaluation of the selected CTC/NFD-OF gel

Various *in-vivo* investigations were conducted to assess the safety, tolerability (Draize test and histopathological examination) and corneal penetration of the prepared CTC/NFD-OF gel (Confocal laser scanning microscopy), as well as its pharmacological activity via pharmacodynamic studies.

Animals used were male adult albino rabbits, with an average weight of ∼2.5 kg. Rabbits were individually housed in cages maintained at 25◦C ± 2 ◦C, with an alternating 12-h light/dark cycle. They were provided with standard commercial food and tap water. Before the commencement of the study, a preliminary examination of all rabbits’ eyes was conducted, and only those without any signs of ocular inflammation were included. This research was approved by the Research Ethics Committee (REC) of the Faculty of Pharmacy, Cairo University, with approval number: PI 3855. We have adhered to the guidelines outlined in the Guide for Care and Use of Laboratory Animals, as published by the US National Institute of Health (NIH Publication No. 85–23, revised 2011).

##### 2.2.14.1 Safety assessment of CTC/NFD-OF gel: Draize test and histopathological evaluation

The Draize test was employed to visually evaluate the ocular irritancy of the sterile CTC/NFD-OF gel (Draize et al., 1944). Before the commencement of the experiment, all animals were examined visually to confirm the absence of ocular redness, irritation, or inflammatory signs. Male albino rabbits (n = 6, average weight ∼2.5 kg) were given 100 μL of the sterile gel into the lower cul-de-sac of one eye (right), while the other eye (left) was kept as a control, receiving normal saline. The CTC/NFD-OF gel was instilled every 2 h for 8 h/day for three successive days (Tawfik et al., 2026). Eyes were inspected at each time point of instillation, and redness or any other signs of irritation were recorded according to a scoring system from 0 (no irritation) to 5 (high irritation). Afterwards, histopathological assessment was conducted to evaluate any potential histopathological alterations in ocular tissues following topical administration of the sterile CTC/NFD-OF gel. At the end of the experimental period, the animals were anaesthetized by intramuscular administration (IM) of Ketamine (200 mg/kg) and xylazine (20 mg/kg) (Ahmed et al., 2026; Hassan et al., 2024). Following IM administration, animals were monitored until adequate anesthetic depth was achieved, indicated by loss of the righting reflex and absence of response to appropriate external stimuli and subsequently euthanized by cervical dislocation, performed by trained personnel, in accordance with the American Veterinary Medical Association (AVMA) guidelines, 2020. The eyes were then excised, rinsed with phosphate-buffered saline (pH 7.4), and fixed in 10% (v/v) formalin saline solution till further processing. The collected ocular tissues were subsequently dehydrated through a serial concentration of alcohol, cleared in xylene, and embedded in paraffin at 56 °C ± 0.5 °C for 24 h. Samples were stained with hematoxylin and eosin (H&E stain), mounted on glass slides, and examined under a light microscope (Leica Imaging Systems Ltd., Cambridge, England) (Ahmed et al., 2025c).

##### 2.2.14.2 Confocal laser scanning microscopy

Confocal laser scanning microscopy (CLSM) was employed to visualize corneal tissues and assess the capacity of the investigated formula to enhance drug permeation without inducing mechanical disruption of the corneal layers (Albash et al., 2021; Oaku et al., 2025). To monitor intrastromal penetration, NFD in the CTC/NFD-OF gel was substituted with 0.1% w/w rhodamine B (RhB) as a fluorescent agent. A volume of 100 μL of the RhB-loaded optimized hyalurosomal gel was instilled into the right eye of a male albino rabbit, while an aqueous RhB solution, instilled in the other eye, served as a control. Following 6 h of ocular administration, the rabbit was anaesthetized and subsequently euthanized as described in the previous Section 2.2.14.1. The corneas were carefully excised, washed with normal saline, preserved in artificial tear solution, and imaged on the same day. Corneal imaging was carried out using an inverted CLSM system (LSM 710; Carl Zeiss, Jena, Germany), equipped with an argon laser, having an excitation wavelength of 485 nm, in addition to a helium-neon laser with an excitation wavelength of 595 nm. The CLSM system is coupled with LSM software (version 4.2, Carl Zeiss MicroImaging, Jena, Germany) (Ahmed et al., 2025c). Image acquisition commenced at the epithelial surface of the cornea, employing z-stack scanning in the xy and xz planes, with sequential optical sections captured until RhB fluorescence was no longer detectable.

##### 2.2.14.3 Effect of the CTC/NFD-OF gel on intraocular pressure (IOP)

A single-dose crossover design was implemented to investigate the effect of CTC/NFD-OF gel and the CTC and NFD aqueous dispersion on IOP, compared to the market eye drops Xola™ (2% Dorzolamide). Six healthy male normotensive albino rabbits, with a mean baseline IOP of 21.3 ± 1.4 mm Hg, were involved in the study. Treatments were applied locally to the lower eyelid of each rabbit’s right eye, while the left eye was set as a control. In the first treatment period, rabbits received the three treatments: CTC and NFD aqueous dispersion, the selected CTC/NFD-OF gel, or Xola™, and the IOP was measured at different time intervals up to 8 h using a calibrated Schiötz Tonometer (Rudolf Riester GmbH and Co. KG, Germany), and the mean IOP of duplicate measurements at each time was recorded. After a 1- week washout period, the IOP of each rabbit’s eyes was measured to confirm that it had returned to its initial baseline value. The procedure was then repeated over two additional treatment periods, each separated by a 1-week washout interval. Treatments were rotated among the rabbits in a crossover manner so that each rabbit received each of the three treatments during the study. The % decrease in IOP was determined as represented in Equation 10 (Tawfik et al., 2026; Fouda et al., 2018):

$$\%\text{ Decrease in IOP}=\frac{IOP\;control\;eye-IOP\;dosed\;eye}{IOP\;control\;eye}$$ (10)

The pharmacodynamic variables studied were the maximum percentage decrease in IOP (Max. % IOP decrease), time to achieve Max. % IOP decrease (t_max_), area under the percentage decrease in IOP versus time curve (AUC_0–8 h_ and ${AUC}_{0-\infty }$ ), and mean residence time (MRT). These variables were quantified using kinetica® 2000 software (Innaphase Corporation, Philadelphia, PA, USA), then statistically analyzed by SPSS® software (Version 25, IBM Corp., Armonk, NY, USA), as a parametric cross-over ANOVA, followed by a post Hoc Duncan’s test (Hande et al., 2019), was employed for the Max % IOP decrease, AUC_0–8h_, ${\text{AUC}}_{0–\infty },$ and MRT, while a non-parametric Friedman test followed by Wilcoxon signed-rank test was used to analyze the t_max_. A P value < 0.05 was considered statistically significant for all analyses (Saher et al., 2023; Daniele et al., 2026).

## 3 Results and discussion

NFD-HAs were successfully fabricated adopting a D-optimal statistical design. Seventeen formulae were evaluated for EE, PS, PDI, and ZP to develop an optimized formula, to which CTC was added, followed by further characterization and evaluation.

### 3.1 *In-vitro* characterization of NFD-HAs

#### 3.1.1 Model analysis of EE (%)

A vesicular system, for ocular administration, should have high EE, which is an imperative feature to circumvent corneal intricacies and foster better absorption and bioavailability (Abdelbary et al., 2016). The calculated EE of the prepared NFD-HAs ranged from 15.9% ± 0.6% to 84.0% ± 1.3%, as shown in Table 2. ANOVA showed that only A: PC amount, as a main effect (not involved in any interactions), had a significant positive effect on EE (*P* < 0.0001), as summarized in Table 3, and shown in Figures 1A,B, denoting that increasing PC amount from 100 to 300 increased EE of NFD. The direct proportional relationship between PC amounts and the EE of NFD can be unswervingly linked to an increase in the number of vesicles formed, besides the increase in their PS due to aggregate formation (which will be confirmed by the PS model analysis) (Saha et al., 2023; Ibrahim et al., 2020). Another perspective suggested that increasing PC, which is lipophilic in nature, makes the environment preferable for the lipophilic drug NFD, log P = 2.5 (Van Der Lee et al., 2001), to settle rather than escape to the external aqueous medium (Elsayed et al., 2025). Many relevant findings support this finding, where Naguib et al. (Naguib et al., 2021), Elsayed et al. (Elsayed et al., 2025), and Makled et al. (Makled et al., 2024) found that the increment of PC amounts in different types of vesicles triggered EE elevation of Ganciclovir, Atorvastatin, and Melatonin, respectively.

**TABLE 3:** Regression coefficients, model type and model evaluation parameters together with related significant model terms.

Responses	Model type	R^2^	Adjusted R^2^	Predicted R^2^	Adequate precision	RSME	Significant model terms
EE (%)	Reduced linear	0.8085	0.7811	0.735	11.5543	13.28	A
PS (nm)	Reduced cubic	0.9999	0.9998	0.9788	373.7968	5.05	A, C, AB, AC, BC, A^2^, B^2^, A^2^B, A^2^C, AB^2^
PDI^1.21	Reduced cubic	1	1	0.9955	832.7619	0.001	A, C, AB, AC, BC, A^2^, B^2^, ABC, A^2^B, A^2^C, AB^2^

* RSME , root square mean error, A = phosphatidyl choline amount, B = edge activator amount, and C = edge activator type.

**FIGURE 1:**
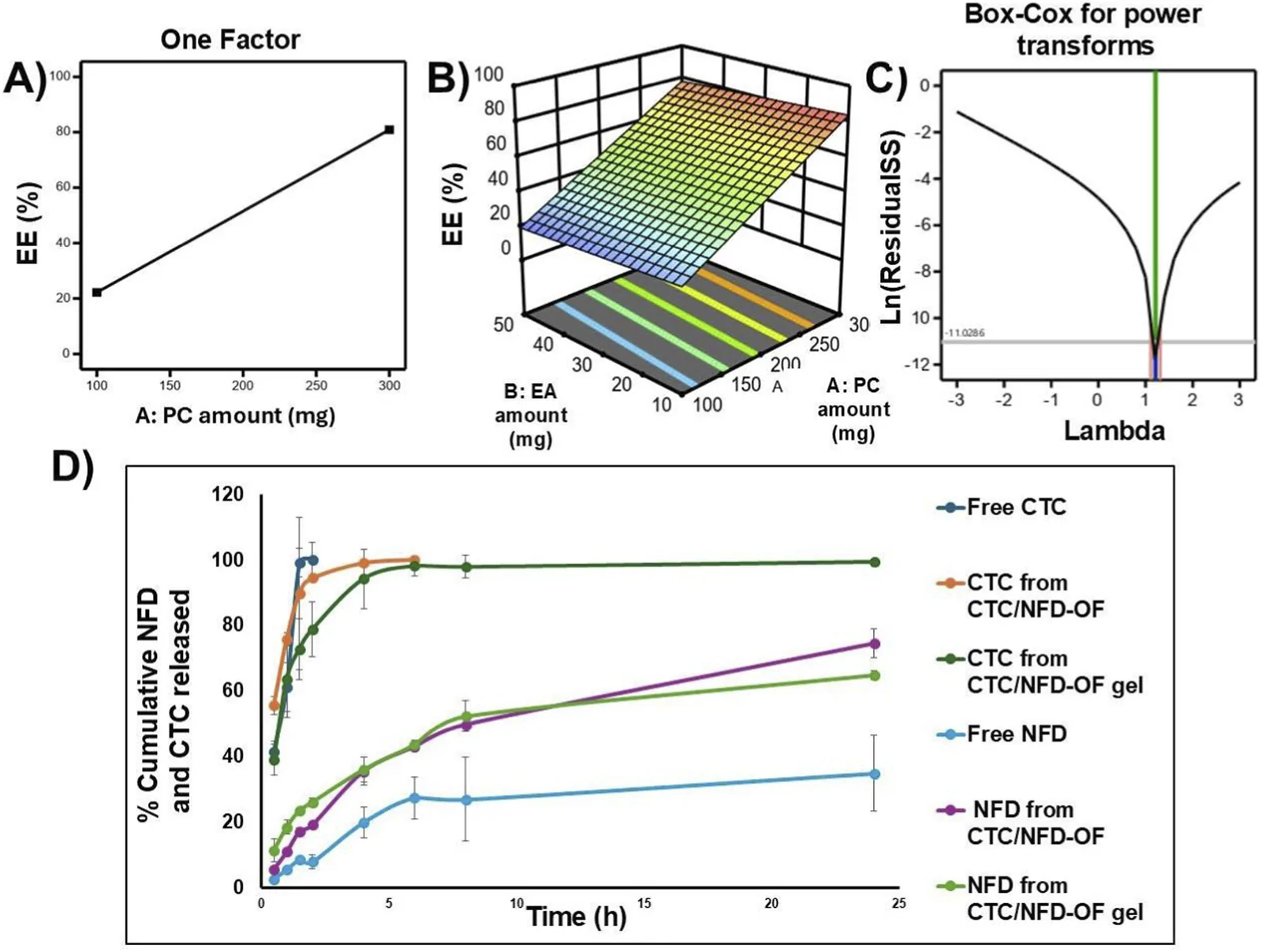
**(A)** A chart showing the main effect of PC amount on the EE% and **(B)** its corresponding 3D surface plot, **(C)** a Box-Cox transformation plot for PDI (raised to power 1.21), as recommended by Design Expert®, and **(D)** Release profiles of CTC and NFD from aqueous solution/suspension, CTC/NFD-OF and CTC/NFD-OF gel.

#### 3.1.2 Model analysis of PS

PS of nanosystems is a very valuable characteristic, which controls the pharmacokinetics, bioavailability and even the toxicity of the loaded drugs (Fan et al., 2020; Li and Huang, 2008). The PS of the prepared NFD-HAs oscillated between 160.8 nm ± 0.6 and 1743.9 nm ± 84.1, as shown in Table 2. The model elucidated showed a significant p-value (<0.0001), with acceptable R^2^, Adjusted R^2^ and prediction R^2^ values, shown in Table 3. ANOVA showed that both A: PC amount and C: EA type had significant effects on the PS, as shown in Table 3. Although B: the EA amount did not appear in the final reduced cubic model as a significant main effect, it was involved in significant interactions with both A and C. Therefore, the effect of the EA amount (B) will be discussed in the context of the AB and BC interaction terms.

Figure 2 represents the AB interaction, as interaction and 3D plots, at the two levels of C: EA type; CO, Figures 2A,B, and DEO, Figures 2C,D. AB interaction at both C levels led to PS enlargement (positive synergistic effect). Increasing the amount of PC, in the presence of either CO or DEO, certainly increases PS. This can be attributed to the higher lipid content, which promotes the formation of thicker bilayers and larger vesicular structures due to increased viscosity and vesicle fusion tendency (Saha et al., 2023; Shaker et al., 2017; Chen et al., 2016).

**FIGURE 2:**
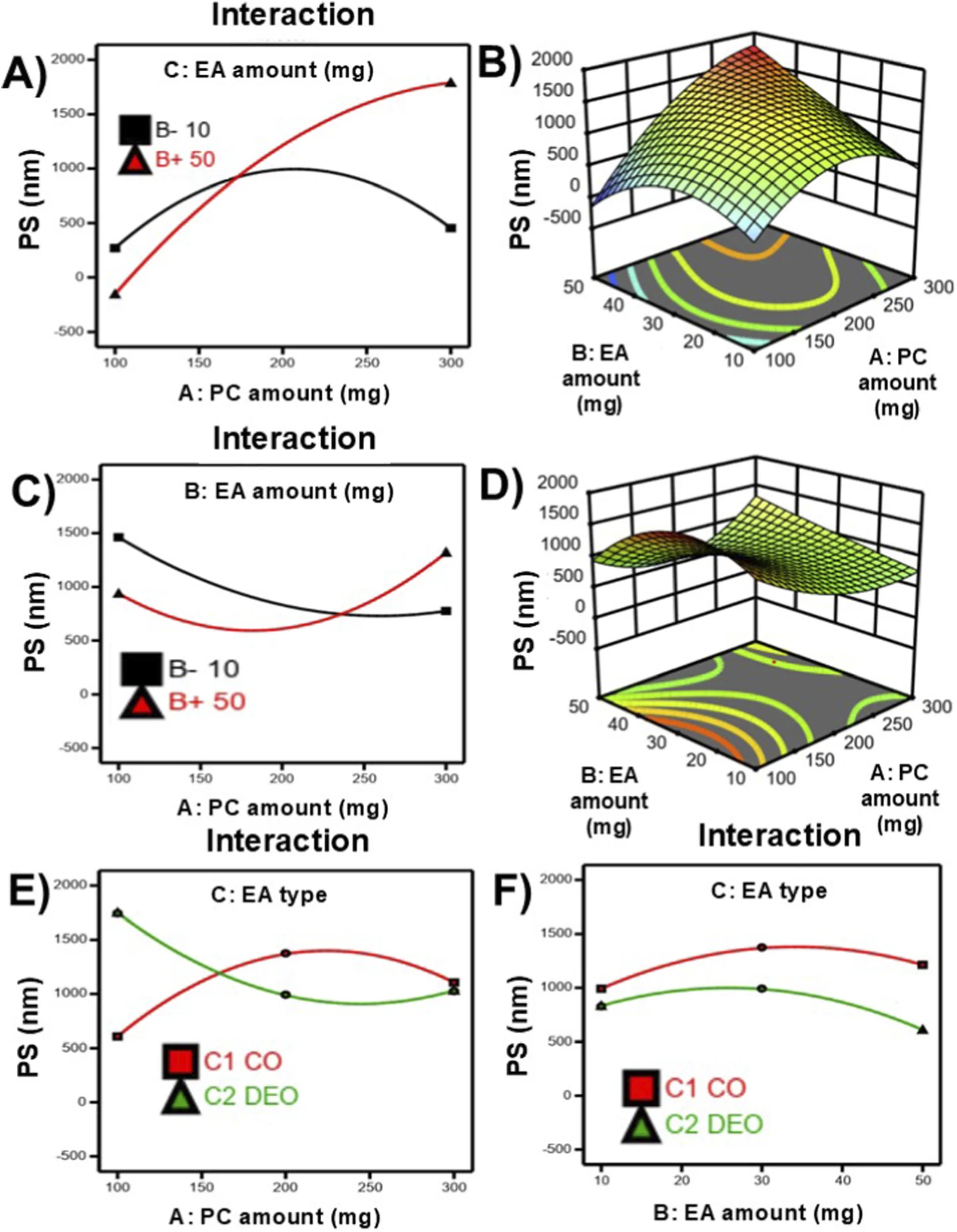
**(A)** Interaction plot showing the effect of **(A)** PC amount and **(B)** EA amount on the PS in presence of CO, along with **(B)** 3D surface plot **(C)** An interaction plot showing the effect of **(A)** PC amount and **(B)** EA amount on the PS in presence of DEO, along with **(D)** its corresponding 3D surface plot, **(E)** An interaction plot showing the effect of **(A)** PC amount and **(C)** EA type, and **(F)** An interaction plot showing the effect of **(B)** EA amount and **(C)** EA type.

For the AC interaction, at an average EA amount (B) of 30 mg (Figure 2E), DEO generated NFD-HAs with less PS than CO, indicating an antagonistic interaction. DEO, being more hydrophobic than CO and possessing stronger membrane-disrupting properties, tends to produce smaller PS, especially at higher concentrations (Kassem et al., 2018). This is likely due to its ability to increase bilayer flexibility and reduce interfacial tension, facilitating the formation of smaller and more deformable vesicles (Elsayed et al., 2025; Sukmawati et al., 2018). CO, which is relatively less hydrophobic, results in larger PS compared to DEO at equivalent concentrations (Lee et al., 2005). This may be explained by its milder effect on bilayer fluidization and lower capacity to prevent vesicle aggregation. BC interaction, at an average PC amount (A) of 200 mg, was depicted in Figure 2F. It shows an antagonistic effect, where the increment in EA amount was accompanied by PS reduction, especially when the EA type (C) is DEO. These observations can be attributed to the ability of EA to fluidize the hyalurosomal membrane, resulting in hyalurosomal size shrinkage. Additionally, increasing EA amounts can lead to micelle formation, which possesses less PS (Nour et al., 2017). These interactions highlight that a balance between PC and EA is essential to acquire a minimum PS.

#### 3.1.3 Model analysis of PDI

PDI is a measure of dispersion homogeneity. A nanosystem should possess a PDI value approaching zero (Danaei et al., 2018). PDI of NFD-HAs ranged from 0.27 ± 0.2 to 0.92 ± 0.1, as listed in Table 2. For the PDI model analysis, a Box-Cox transformation was recommended and applied to PDI values by DX13. All PDI values were raised to the power of 1.21, see Figure 1C. The Box–Cox transformation is a statistical tactic to slightly adjust the distribution of the response data into a more normal distribution; thus, the variance becomes more constant, enabling better model fitting and reliable statistical analysis (Box and Cox, 1964). A significant reduced cubic model was obtained (*p < 0.0001*), with an ideal R^2^, Adjusted R^2^ and prediction R^2^, as shown in Table 3. The three interaction terms AB, AC, and BC showed significant effects (*p < 0.0001*), as depicted in Figure 3. The interaction between A: PC amount and B: EA amount at the low level of C: EA type (CO), Figures 3A,B, showed a pronounced increase in PDI, while, in case of the high level of C: EA type (DEO), Figures 3A,C,D slight elevation in PDI was observed. This can be related to the PS elevation assisted by increasing PC amounts, triggering viscosity elevation and hindering the formation of uniform PS (Masarudin et al., 2015). Similarly, a positive AC interaction was detected as shown in Figure 3E, signifying that NFD-HAs prepared from PC and DEO possessed higher PDI than those prepared from PC and CO. This may be because the hydrophobicity of DEO is greater than that of CO, magnifying its interaction within the phospholipid bilayer, and this may induce irregular packing of PC, resulting in a broader PS range and subsequently higher PDI. In other words, EAs with higher HLB (e.g., CO) were reported to produce vesicular systems with more uniform PS (Bnyan et al., 2018; Chaithra et al., 2025). On the other hand, it was found that the interaction between B: EA amount and C: EA type had a negative effect on PDI, as illustrated in Figure 3F. This means that increasing the EA amount led to a significant decline in PDI (which is favorable). Given that EA plays an important role in preserving consistent PS, a decrease in PDI is an expected consequence, in addition to being a solubilizing agent (Bnyan et al., 2018).

**FIGURE 3:**
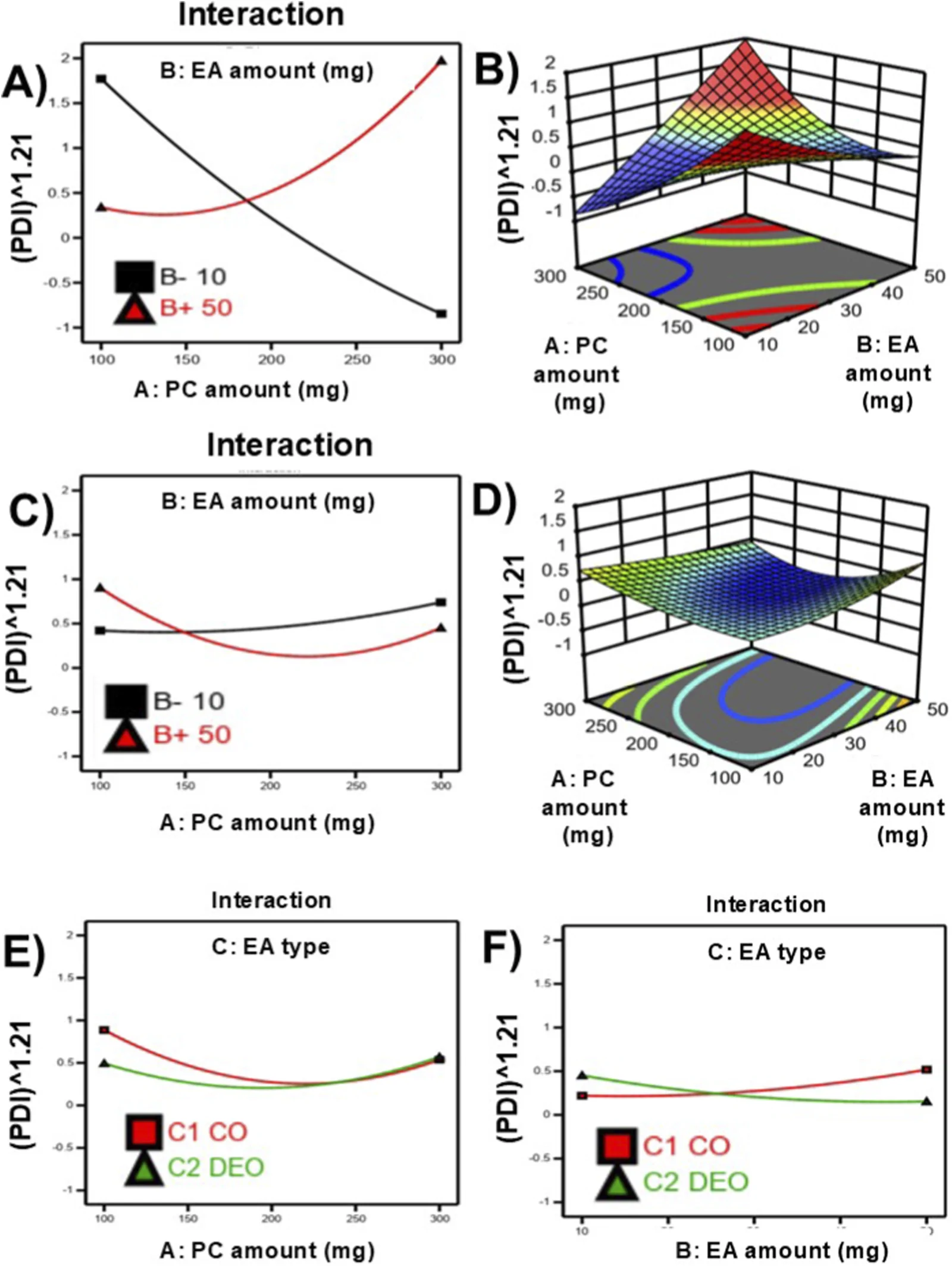
**(A)** Interaction plot showing the effect of **(A)** PC amount and **(B)** EA amount on the PDI in presence of CO, along with **(B)** its 3D surface plot **(C)** An interaction plot showing the effect of **(A)** PC amount and **(B)** EA amount on PDI in presence of DEO, along with **(D)** its corresponding 3D surface plot, **(E)** An interaction plot showing the effect of **(A)** PC amount and **(C)** EA type on PDI, and **(F)** An interaction plot showing the effect of **(B)** EA amount and **(C)** EA type.

#### 3.1.4 ZP

The vesicles’ surface charge is known as ZP. Higher ZP is required to thwart particle aggregation through electric repulsion, so it is considered an indirect reflection of system stability (Ahmed et al., 2025c). Through literature review, it was found that nanosystems should exhibit a ZP value ≥ 20 mV as an absolute value to endow acceptable stability (Sharma and Hector, 2026; Ahmed et al., 2025d). Herein, the ZP of the prepared NFD-HAs is between −28.0 ± 4.1 mV and −45.6 ± 1.8 mV, as shown in Table 2, indicating that the prepared NFD-HAs are expected to exhibit reasonable stability. It is noteworthy that all formulae exhibited negative ZP due to the intrinsic negative charges originally present on PC and the anionic bile salts (Kassem et al., 2018; El, 2017).

### 3.2 NFD-HA optimization and evaluation of NFD-OF

An optimized formula (NFD- OF) was selected by DX13, having a desirability of 0.905 with an importance equal to (3+) designated to EE, PS, and PDI. The composition of NFD-OF suggested by DX13 was 300 mg PC and 10 mg of CO, and the proposed values for the characterized variables were 83.8% for EE and 454.5 nm for PS. After evaluating the measured responses for NFD-OF, the EE was quantified as 83.7% ± 1.4, and the PS was measured to be 436.4 nm ± 32.0, with a PDI of 0.4 ± 0.1. The NFD-OF was further sonicated for 40 s by a probe sonicator (Model UP-500, 220V, ChromTech, Apple Valley, Minnesota, USA) to decrease PS and PDI to support the *in-vivo* corneal penetration for better glaucoma control. An additional after-sonication re-evaluation was performed, yielding a reasonable EE (70.7% ± 1.8%) and adequately low PS and PDI (198.6 ± 23.1 nm and 0.4 ± 0.1, respectively). While ZP was reduced from −40.9 ± 1.4 mV to −39.1 ± 1.3 mV, which is still acceptable, maintaining the dispersion’s repulsion and physical stability.

### 3.3 *In-vitro* drug release from CTC/NFD-OF and its corresponding gel

The release of CTC from CTC/NFD-OF gel was biphasic, with an initial quick release (∼40% at 0.5 h) followed by a period of slow but sustained release up to 24 h. While NFD showed a more controlled release over the time of the experiment, without a sharp initial phase, Figure 1D. By investigating the release of the two drugs from CTC/NFD-OF, similar but more rapid release profiles were obtained. This is exactly related to the increased viscosity of the gel. Comparing the release results of CTC/NFD-OF and its corresponding gel to the CTC aqueous solution release results showed that the CTC solution released ∼100% of CTC in 1 h, while the NFD suspension release results showed that the NFD suspension released only 2.4% in the first ½ h and about 9.4% in the next 2 h. Upon inspecting these results, it was concluded that CTC/NFD-OF gel sustained the release of both drugs in a manner appropriate to the nature of each drug, as it enhanced the release of the poorly water-soluble NFD (compared to its suspension), while prolonging the release of a highly water-soluble drug like CTC (compared to its solution), thereby achieving adequate drug release to assure the intended therapeutic purpose.

Regarding the release kinetics, both drugs exhibited first-order release from the CTC/NFD-OF and the CTC/NFD-OF gel. By comparison, the zero-order model had lower R^2^ values for both CTC and NFD, as shown in Table 4, indicating that the drugs’ release was concentration-dependent. In other words, the release was more rapid at the beginning due to the higher drug concentration within the formulae and gradually decreased over time as the drug content declined (Shafiei et al., 2021). While NFD release from NFD suspension also showed a first-order release pattern, interestingly, CTC release from its aqueous solution was best fitted to the zero-order kinetic model, indicating an approximately constant release over time. This behavior may be attributed to the high aqueous solubility of CTC, together with the maintenance of sink conditions and continuous diffusion across the dialysis membrane.

**TABLE 4:** Kinetics analysis of the release profiles of Citicoline sodium and Nifedipine from the CTC/NFD-OF, its corresponding gel, alongside Nifedipine suspension and Citicoline solution.

Formulae	Zero order	First order	Korsmeyer-Peppas	(n) Exponent for Korsmeyer-Peppas
	R^2^	R^2^	R^2^	
CTC from CTC solution	0.9049	0.8094	0.9528	0.6913
CTC from CTC/NFD-OF	0.6587	0.9881	0.8797	0.2843
CTC from CTC/NFD-OF gel	0.7099	0.9301	0.8802	0.3164
NFD from NFD suspension	0.9294	0.9343	0.9743	0.8595
NFD from CTC/NFD-OF	0.9618	0.9844	0.986	0.7782
NFD from CTC/NFD-OF gel	0.9665	0.9875	0.9902	0.5249

As for the Koresmeyer-Peppas model applied for the determination of the release mechanism of the two drugs, the R^2^ for all release profiles were high, indicating data fitting to the model, see Table 4. The exponent (n) values reflect the mechanism of release; when n is ≤ 0.5, the release follows a Fickian diffusion mechanism, but when 0.5 < n < 1.0, this refers to a non-Fickian transport mechanism (Ojsteršek et al., 2024). CTC release profiles from CTC/NFD-OF and CTC/NFD-OF gel showed n values < 0.5 (0.2843 and 0.3164, respectively), indicating Fickian diffusion-controlled release. This is due to the diffusion of CTC through the aqueous external phase and the gel matrix. NFD release from NFD suspension showed a release pattern managed by a non-Fickian transport mechanism, n > 0.5. This release mechanism is characteristic of water-insoluble drugs, like NFD, from their suspensions (Gao et al., 2021). NFD release from CTC/NFD-OF and CTC/NFD-OF gel was also governed by a non-Fickian transport mechanism. The non-Fickian release behavior of NFD from CTC/NFD-OF and CTC/NFD-OF gel suggests that NFD release is governed by a combination of diffusion and polymer erosion, swelling, and relaxation mechanisms (Fu and Kao, 2010). This finding highlights the ability to modulate the drug release through cautious optimization of formulation variables and hydrogel properties. Such control over the release mechanism is advantageous for the development of sustained drug delivery systems, capable of preserving therapeutic drug levels for prolonged periods, thereby potentially enhancing treatment efficacy, reducing dosing frequency, and improving patient adherence (Elsayed et al., 2025).

### 3.4 Transmission electron microscopy (TEM)

TEM serves as an indispensable tool for discerning morphology and measuring the vesicular diameter of meticulously crafted nanostructures (Malatesta, 2021). CTC/NFD-OF showed a well-formed round structure, as shown in Figure 4A, with a multilamellar hyalurosomal gel core clearly recognizable in Figure 4B. Additionally, the TEM image represented in Figure 4C showed the CTC/NFD-OF gel after sonication and sterilization (PS ∼180 nm), confirming the conserved vesicular membrane integrity and small PS.

**FIGURE 4:**
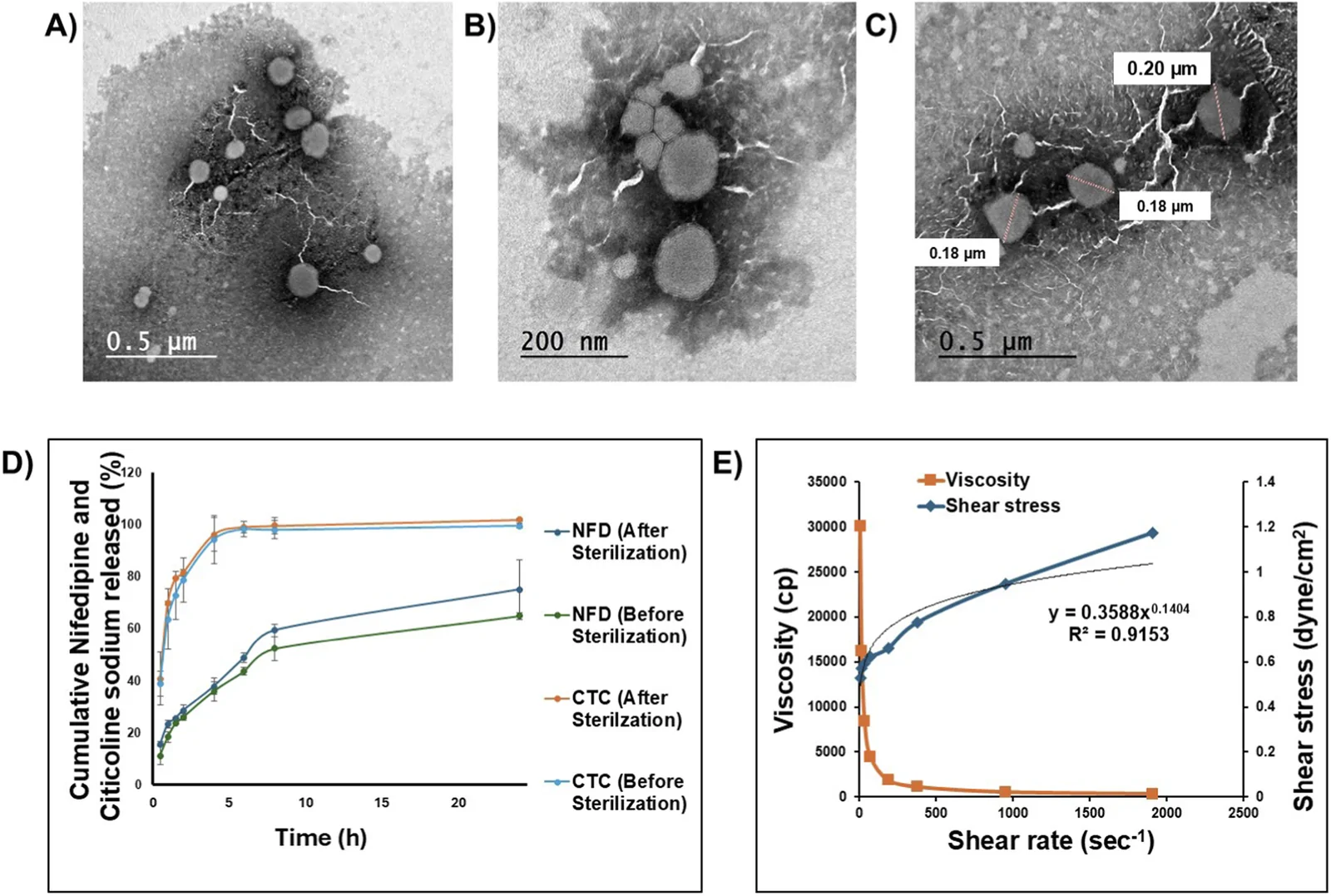
**(A)** Transmission electron micrographs (TEM) of CTC/NFD-OF, **(B)** TEM showing multilamellar hyalurosomes with gel core, **(C)** TEM of the sonicated CTC/NFD-OF gel after sterilization, **(D)** the effect of terminal sterilization on the release profile of CTC and NFD from CTC/NFD-OF gel, and **(E)** the rheological behavior of CTC/NFD-OF gel.

### 3.5 FTIR spectra analysis

FTIR spectra were obtained for individual components (phosphatidylcholine, Sodium cholate, HA, NFD, CTC, HPMC) as well as for the lyophilized CTC/NFD-OF gel, as shown in Figure 5. By analyzing the FTIR chart of PC, Figure 5A, a peak at 1733 cm^-1^ (carbonyl ester, C=O stretching), a peak at 1,465.9 cm^-1^ (hydrocarbon chains, CH_2_ scissoring), peaks at 1,238.3 and 1,091.7 cm^-1^ for the phosphate group (C–O–P) symmetric and asymmetric stretching, respectively, and the choline head C-N stretching peak at 972.12 cm^-1^, were observed (Ghyadh and Al-Khedairy, 2023; Süleymanoglu, 2009).

**FIGURE 5:**
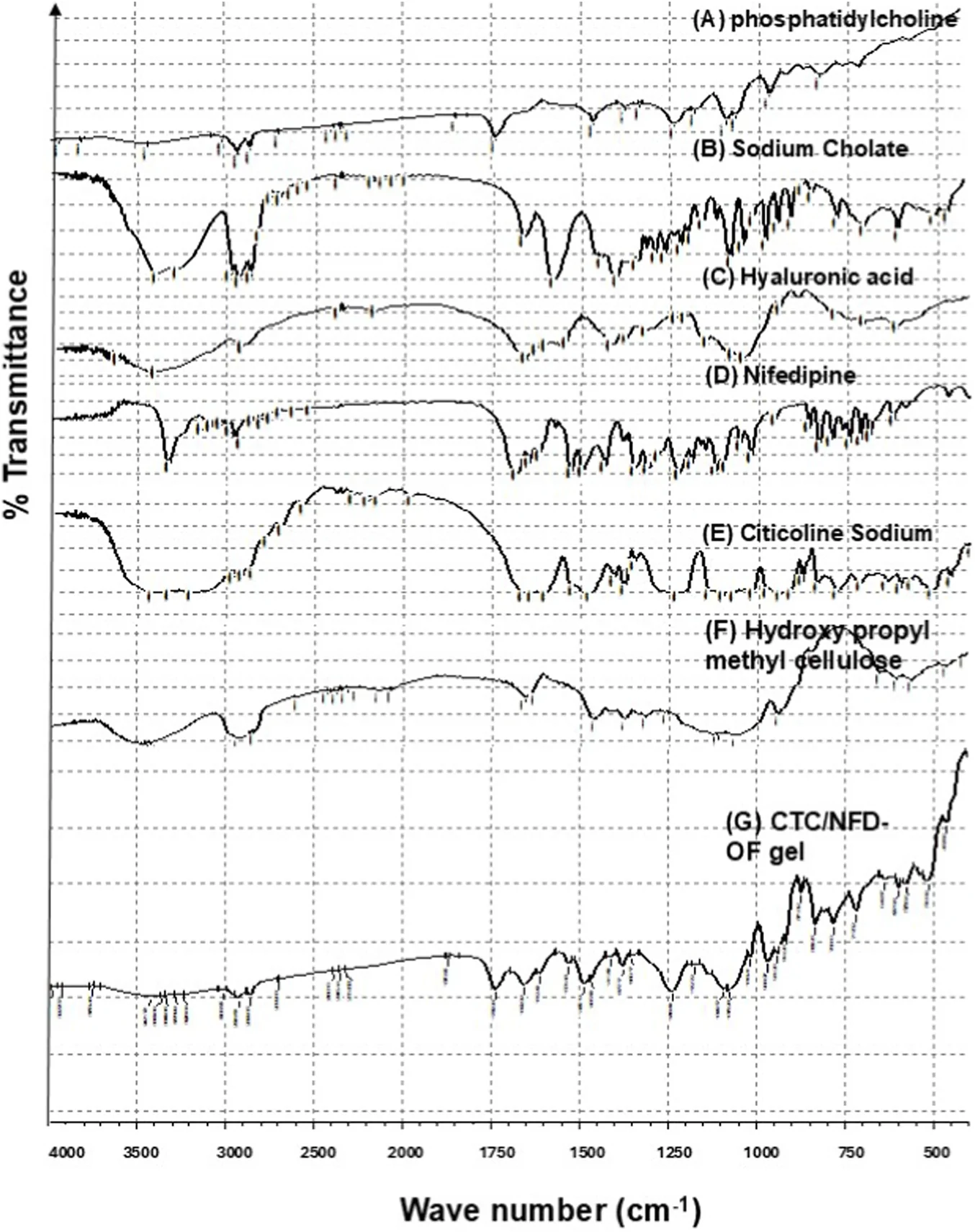
FTIR spectra of **(A)** phosphatidylcholine, **(B)** Sodium cholate, **(C)** Hyaluronic acid, **(D)** Nifedipine (NFD), **(E)** Citicoline sodium (CTC), **(F)** HPMC, and **(G)** CTC/NFD-OF gel.

The FTIR chart of sodium cholate (CO), Figure 5B, shows the OH stretching at (3,700–3,000 cm^−1^), aliphatic C-H stretching of the steroid backbone at 2,970, 2,933 and 2,862 cm^-1^, and C=O stretching band of COOH at 1,581 cm^−1^ (Kamel A et al., 2020). Upon examining the FTIR chart of HA, Figure 5C, a peak at 3,406 cm^-1^ (OH and NH stretching), a peak at 2,920 cm^-1^ (stretching of C-H), a peak at 1,654 cm^-1^ (amide carbonyl (C=O) stretching) and a peak at 1,419 cm^-1^ (COO- stretching), referring to the acid group of molecule HA, were detected. Additionally, an absorption band at 1,045 cm^-1^, corresponding to C-O-C bond stretching, was observed (Eladawy et al., 2021). The FTIR chart of NFD, Figure 5D, shows a sharp characteristic peak at 3,329 cm^-1^ (N-H stretching), a peak at 3,082 cm^-1^ (aromatic -CH stretching), a peak at 1,685 cm^-1^ (C=O ester stretching), a peak at 1,624 cm^-1^ (pyridine ring breathing), a peak at 1,527 (nitro group, stretching), and 1,226 cm^-1^ (C-O ester stretching) (Ozon et al., 2022).

The FTIR chart of CTC, Figure 5E, demonstrates O─H and N─H stretching bands at ∼3,200–3,400 cm^-1^ and C─H stretching bands at 2,877, 2,904, and 2,961 cm^−1^. The FTIR spectrum of CTC also exhibited a characteristic peak at 1,600 cm^-1^, denoting obvious (C=O) stretching of the cytosine ring, and a peak at 1,477 cm^-1^, corresponding to the (C=N) of cytosine. Moreover, peaks at ∼1,230 and ∼1,095 cm^−1^, referring to (P=O) stretching of the phosphate group and (C─O) in tetrahydrofuran, respectively, were observed. Additionally, (P─O) stretching peak at ∼ 1,114 cm^-1^ and C-N^+^-C stretching peaks (asymmetric and symmetric of tertiary amine) at ∼975 and 914 cm^-1^ were detected (Bonechi et al., 2023; Sreenivasa Reddy et al., 2013).

The FTIR chart of HPMC, Figure 5F, shows an absorption peak at ∼3,444 cm^-1^, which was assigned to the stretching of the hydroxyl (-OH) group, and two peaks at ∼2,931 and ∼2,839 cm^-1^, referring to C–H stretching of -CH_3_ and -CH_2_, respectively. Peaks at 1,453 and 1,377 cm^-1^, due to the bending of CH_2_ and C-H of the methyl group, in addition to the C–O–C stretching peak, at ∼1,064 cm^-1^, of the glycosidic bond, were also observed. The HPMC peaks observed were similar to those reported by Iqbal et al. (Iqbal et al., 2017).

The FTIR spectrum for the lyophilized CTC/NFD-OF gel, Figure 5G, demonstrates the disappearance of NFD-specific peaks, which may be due to the entrapment of the drug into the prepared hyalurosomes, while some of the CTC-specific peaks are still recognizable, but with attenuated intensity, indicating the integration of CTC into the entangled HPMC gel matrix.

### 3.6 pH, surface tension and refractive index measurements of CTC/NFD-OF gel

It is essential to measure the pH of the CTC/NFD-OF gel to guarantee its suitability for ocular use. The pH of the CTC/NFD-OF gel was 6.9 ± 0.02. Given that the physiological pH of tears is 7.4, the suitability of the CTC/NFD-OF gel for ocular application can be confirmed (Ahmed et al., 2025c). Moreover, Mohanty et al. (Mohanty et al., 2013) reported that a pH value range from 4 to 8 might encourage ocular permeation; therefore, the pH of the CTC/NFD-OF gel is optimal for supporting its efficacy, while conserving its safety. Regarding surface tension measurements, it's crucial to mention that the presence of surfactants (EAs) and PC in the CTC/NFD-OF gel led to a reduction in its surface tension (58.0 ± 0.2 mN/m), compared to water (72 mN/m) (Vicario-de-la-Torre et al., 2014). This is supposed to encourage more corneal penetration, as later confirmed by CLSM, and enhance the therapeutic efficacy (Dave and Paliwal, 2014). Additionally, the reduced surface tension not only enhances corneal penetration but also prolongs the residence time, as it was reported that ocular formulae exhibiting a surface tension close to that of the tear film (44 mN/m (Tiffany et al., 1989)) are supposed to retain more on the cornea (Fouda et al., 2018). This was subsequently supported by the pharmacodynamics study. It is certainly important to avoid blurry vision; thus, refractive index measurement for ocular preparations is of great significance. Ammar et al. (Ammar et al., 2009) reported that a refractive index less than 1.476 would be acceptable for ocular preparations. The refractive index of the CTC/NFD-OF gel was 1.37 ± 0.0 (i.e., <1.476), offering patient comfort and a lack of blurry vision following its topical application.

### 3.7 The effect of terminal gamma sterilization on CTC/NFD-OF gel

It is noteworthy to highlight that CTC/NFD-OF gel was characterized by EE, PS, ZP and PDI after the addition of CTC and the gelling agent (HPMC) to the optimized formula (NFD-OF). A substantial decrease in ZP was observed (from −39.1 ± 1.3 mV to −22.8 ± 1.3 mV), possibly due to the interaction of the positively charged sodium ions of CTC (used as a water-soluble sodium salt) with the negatively charged NFD-OF, in addition to hindering the NFD-OF electrophoretic motion by the viscosity of the formed gel. However, this change still maintains hyalurosomal stability within the gel (≥± 20 mV) (Sharma and Hector, 2026). It was also accompanied by an increase in the PDI value from 0.4 ± 0.1 to 0.670 ± 0.0 due to increased gel viscosity, while the PS and EE remained unchanged.

PS, PDI, EE, and ZP of the CTC/NFD-OF gel were re-evaluated after sterilization to assess the impact of the sterilization process on the characterized gel, in addition to release studies and TEM imaging. Results confirmed the safety and tolerability of the sterilization method used, with no adverse effects observed in the prepared CTC/NFD-OF gel. The PS of the sterilized CTC/NFD-OF gel was 198.1 nm ± 10.6 and 197.3 nm ± 0.8 before and after sterilization, respectively, while maintaining its shape and intact membrane, as verified by TEM in Figure 4C. EE value was consistent, being 70.7% ± 2.3% prior to sterilization and 71.5% ± 0.2% after sterilization. ZP also remained stable, with no notable difference (−22.8 ± 1.3 mV and −22.2 ± 1.6 mV before and after sterilization, respectively). PDI values were similar (0.670 ± 0.0 and 0.674 ± 0.0 before and after sterilization, respectively). Additionally, the similarity factor calculated for the release profiles of both NFD and CTC, each compared to its original profile before sterilization, Figure 4D, was 62.7 for NFD and 71.4 for CTC, confirming the stability of the CTC/NFD-OF gel following exposure to gamma radiation at a dose of 25 KGy.

### 3.8 Rheological properties of the CTC/NFD-OF gel

Rheological properties are valuable attributes in hydrogel preparation and application, as they influence the product’s physical appearance, texture and flow behavior (Iqbal et al., 2017), and accordingly affect patient acceptability and therapeutic efficacy. The CTC/NFD-OF gel was evaluated using a rotational Brookfield viscometer at 25 ± 1^o^C. The rheograms of the CTC/NFD-OF gel are demonstrated in Figure 4E. Results showed that the CTC/NFD-OF gel exhibited a shear-thinning flow as the viscosity dropped with the rise in shear rate. The flow index (n) value of the gel was much lower than one (n = 0.1404). Therefore, the CTC/NFD-OF gel possessed a non-Newtonian shear-thinning behavior, as determined by the power law equation. Similar results were observed by Pan et al. (Pan et al., 2023), describing the flow behavior of HPMC-based hydrogels.

Furthermore, data were best fitted to Carreau’s equation, which yielded the highest coefficient of determination, indicating a pseudoplastic flow, where R^2^ values for Carreau’s, Casson’s, and Bangham’s models were 0.9999, 0.9957, and 0.9629, respectively. The pseudoplastic behavior of the hydrogels carrying nano systems has been commonly reported (Elsayed et al., 2025), and it is interestingly noted that the pseudoplastic flow is highly advantageous for ophthalmic preparations, as, at high shear rates occurring during normal blinking, after application, the viscosity of the hydrogel decreases, offering convenient use, whereas at low shear rates, known as interblinking periods, it retains its normal viscosity and, thereby, it gains more ocular residence (Chen et al., 2023).

The pseudoplastic behavior of hydrogels can be explained by the balance between shear-induced structural changes and the random movement of polymer chains caused by Brownian motion. At rest, Brownian motion maintains the polymer chains coiled, trapping water within their structure. When shear stress is employed, these coiled chains gradually straighten and align in the direction of flow. As the shear rate increases, this alignment predominates, reducing the resistance to flow and consequently lowering the system’s viscosity. Moreover, some of the water associated with the hydrogel polymer chains may be freed during the increment of the shear rate, efficiently decreasing the apparent molecular weight and concentration of their structures (Fahmy et al., 2018).

### 3.9 *In-vitro* antioxidation (scavenging DPPH free radical)

Free radicals and the resulting oxidative stress play a perilous role in the development of glaucoma. They can damage vital ocular cells, including the trabecular meshwork and RGCs, thereby impairing mitochondrial function within cells and potentially leading to progressive neurodegeneration. While physicians recommend oral ingestion of antioxidants, topical CTC solution in a concentration of 2% (as that of CTC/NFD-OF gel) was reported to be beneficial in relieving optic nerve degeneration (Carnevale et al., 2019). The potential antioxidant activity of the CTC/NFD-OF gel may be because of the integral harmony between the role of the two drugs, including CTC antioxidant activity reinforced by the oxidative stress-relieving effect of NFD (Yao et al., 2020; Kang et al., 2010). The *in-vitro* DPPH free radical scavenging assay showed the superiority of the CTC/NFD-OF gel, as the inhibition of free radicals by the CTC/NFD-OF gel showed a value of 16.44 ± 0.7 µg/mL for DPPH-IC_50_ compared to that of NFD (219.43 ± 1.76 µg/mL) and CTC (234.62 ± 4.9 µg/mL) alone, while the blank hyalurosomal gel showed no antioxidant activity (IC_50_ is > 1,000 µg/mL) (Reviana et al., 2021), confirming the antioxidant effect of the two drugs, which reflects their neuroprotection contribution and gives a promising platform for glaucoma treatment support.

### 3.10 *In-vivo* evaluation of the selected CTC/NFD-OF gel

#### 3.10.1 Safety assessment of CTC/NFD-OF gel: Draize test and histopathological evaluation

No redness or inflammation signs were observed in the Draize test, as all-time points’ observations were given a score of zero. Furthermore, upon examination of the stained excised ocular samples, the samples of the control eye received normal saline solution, Figure 6A, as well as those received the CTC/NFD- OF gel, Figure 6B, showed no histopathological alterations with preserved epithelial and stromal structures. These findings entirely verify the acceptability and safety of the CTC/NFD-OF gel to be administered topically in the eyes, without possessing any adverse or deteriorating effect on the ocular tissues.

**FIGURE 6:**
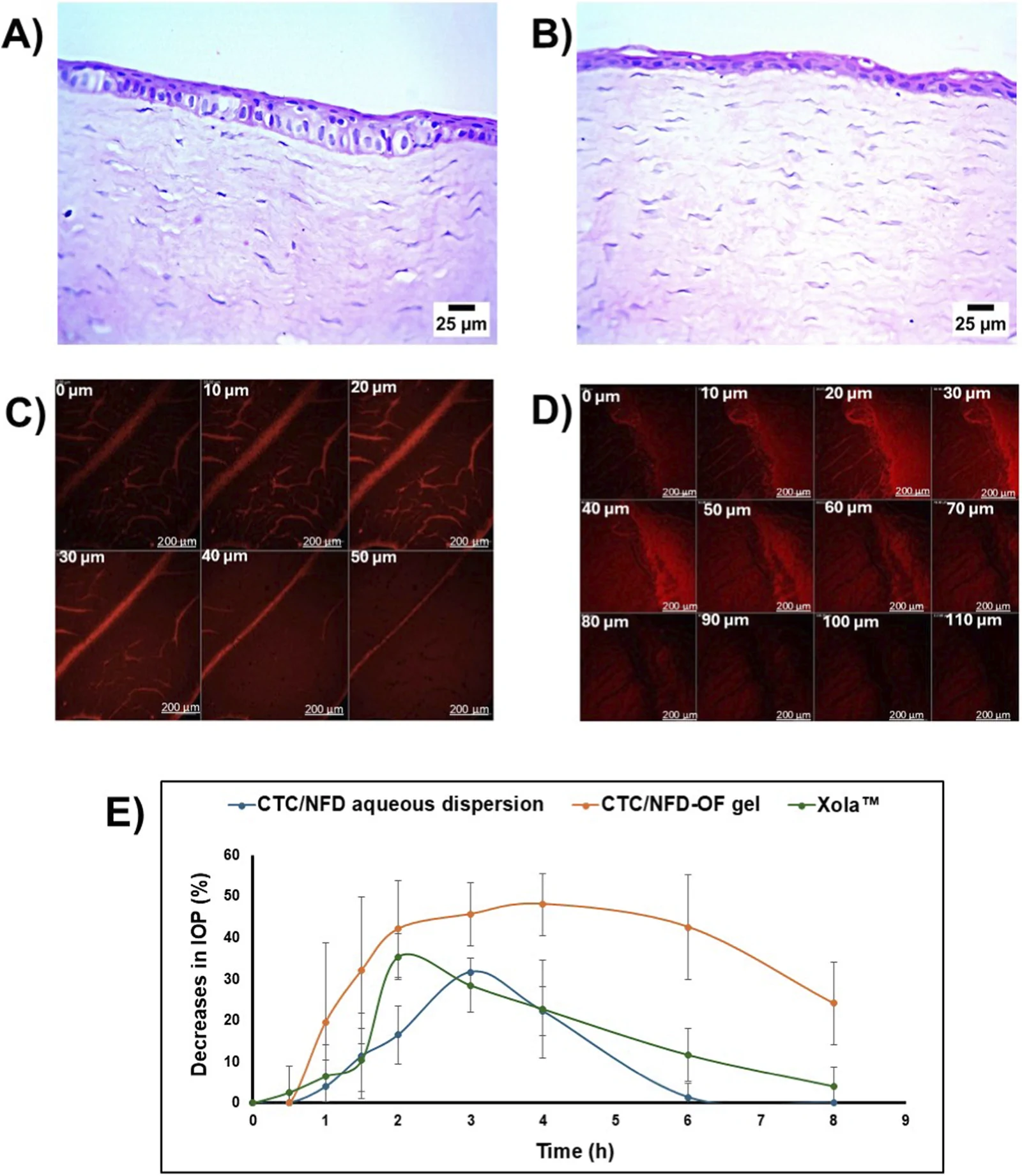
Histopathological sections (epithelium and stroma) of **(A)** rabbit eye treated with normal saline, **(B)** rabbit eye treated with CTC/NFD-OF gel, Confocal laser scanning micrographs of rabbit corneas after topical administration of **(C)** Rhodamine B aqueous solution and **(D)** Rhodamine B-loaded optimized hyalurosomal gel, and **(E)** *In-vivo* decrease in IOP profiles of CTC/NFD aqueous dispersion, CTC/NFD-OF gel compared to Xola™ eye drops (Mean ± SD).

#### 3.10.2 Confocal laser scanning microscopy (CLSM)

CLSM is an objective method used to examine the transcorneal invasion of RhB solution (0.1% w/v) and RhB-loaded optimized hyalurosomal gel after ocular application by tracking the fluorescence radiated by corneal tissues. CLSM can evaluate the ability of the optimized hyalurosomes to facilitate deeper penetration of the loaded drug through the corneal tissues. RhB was selected as a fluorescent probe for the CLSM study because its strong fluorescence permits sensitive visualization of corneal penetration and distribution within ocular tissues (Guss et al., 1984). Although RhB is not identical to NFD, fluorescent probes are commonly employed in CLSM studies when direct imaging of the drug is not applicable due to the lack of intrinsic fluorescence. Being a lipophilic molecule (Vult von Steyern et al., 1996), similar to NFD (Van Der Lee et al., 2001), RhB was loaded into the same nanosystem and subjected to the same formulation conditions as NFD; hence, the observed penetration pattern mainly reflects the delivery behavior of the nanosystem rather than the fluorescent molecule itself. Accordingly, the CLSM findings provide qualitative evidence of the improved corneal penetration potential of the developed nanosystem.

As shown in Figure 6C, RhB solution served as a control, revealing a penetration depth of 50 μm, whereas the optimized hyalurosomal gel achieved penetration up to 110 μm (Figure 6D). This indicates a 2.2-fold increase in corneal penetration facilitated by the nano-hyalurosomal structure, demonstrating its capacity to accomplish its therapeutic goal more effectively.

#### 3.10.3 Assessment of the pharmacological activity of CTC/NFD-OF gel

The study protocol was approved by the “Research Ethics Committee for experimental and clinical studies at Faculty of Pharmacy, Cairo University” (PI #3855), and it adheres to EU Directive 2010/63/EU for animal experiments.

The % decrease in IOP effect of the three treatments is illustrated in Figure 6E, and the assessed pharmacodynamic parameters (mean ± SD) are listed in Table 5. AUC_0–8 h_, and ${\text{AUC}}_{0–\infty }$ reflect the magnitude of drug exposure to the last definitely measured time (8 h) and to the extrapolated to infinite time, respectively, while Max. % IOP decrease measures the extent of drug efficacy, t_max_ reflects the time to reach the Max. % IOP decrease, and MRT indicates the time of drug exposure (Duan, 2010). A marked increase in the Max. % IOP decrease, MRT, AUC_0–8 h_, and ${\text{AUC}}_{0–\infty }$ of the CTC/NFD-OF gel, supported by various statistical evaluations, was observed. ANOVA results, followed by a *post hoc* Duncan’s test, for the Max. % IOP decrease, AUC_0–8 h_, ${\text{AUC}}_{0–\infty }$ , and MRT showed a significant increase in the pharmacodynamic parameters of the CTC/NFD-OF gel (51.8% ± 8.2%, 264.8 ± 49.9, 494.1 ± 191.9, and 8.9 ± 2.7 h, respectively) compared to those of the CTC/NFD dispersion and Xola™. On the other hand, there was no significant difference between the aqueous dispersion and Xola™, where the Max. % IOP decrease, AUC_0–8 h_, ${\text{AUC}}_{0–\infty }$ , and MRT of the CTC/NFD dispersion were 31.8% ± 3.3%, 82.7 ± 39.2, 85.5 ± 30.5, and 3.8 ± 1.6 h, respectively, and those of Xola™ were 36.4% ± 5.9%, 123.7 ± 32.6, 182.8 ± 33.7, 4.1 ± 0.8 h, respectively. The same findings were confirmed by the Friedman test, followed by the Wilcoxon signed-rank test, for the t_max_ parameter, which showed that the t_max_ of the CTC/NFD-OF gel (4.3 ± 0.8 h) was significantly higher than that of the dispersion (3.0 ± 0.0 h) and Xola™ (2.8 ± 1.0 h), while the t_max_ of the dispersion and Xola™ were not significantly different from each other.

**TABLE 5:** Non-compartmental pharmacodynamic parameters of CTC/NFD-OF gel compared to those of CTC and NFD dispersion, expressed as (mean ± SD).

Pharmacodynamic parameters	CTC/NFD-OF gel	CTC/NFD dispersion	Xola™	*p*-value (significance)^a^
Max. IOP decreased (%)	51.8 ± 8.20	31.8 ± 3.30	36.4 ± 5.90	0.005
^b^T_max_ (h)	4.30 ± 0.80	3.00 ± 0.00	2.80 ± 1.00	0.016
$\text{AU}C_{0-8\;h}$	264.8 ± 49.9	82.7 ± 39.2	123.7 ± 32.6	0.000
$\text{AU}C_{0-\infty }$	494.1 ± 191.9	85.5 ± 30.5	182.8 ± 33.7	0.001
$\text{MRT }\left(h\right)$	8.90 ± 2.70	3.80 ± 1.60	4.10 ± 0.80	0.001

^a^ Significance at p < 0.05.

^b^ Non-parametric Friedman Test.

These findings provide evidence of an enhancement in the ocular hypotensive pharmacological activity of the CTC/NFD-OF gel due to several factors: (1) the strong penetrating ability of NFD-loaded hyalurosomes, supported by their lipophilicity and nano-scaled size (this was verified by CLSM); (2) the longer residence time (MRT) of the CTC/NFD-OF gel, owing to its rheological properties; (3) the intrinsic characteristics of other components of the CTC/NFD-OF gel, such as CO, which was reported as a solubilizing agent and permeation enhancer (Moghimipour et al., 2015), and the reported tissue hydration and mucoadhesion effects of HA (Guarise et al., 2023); and (4) the sustained release of CTC resulting from HPMC features (as shown in the *in-vitro* release studies). The interaction among these factors may improve the bioavailability of the CTC/NFD-OF gel and, consequently, its pharmacological potential as glaucoma therapy.

## 4 Study limitations

Limitations of the current study include the lack of a pharmacokinetic evaluation following ocular administration. Although CTC/NFD-OF gel was comprehensively characterized and its therapeutic efficiency was demonstrated *in vivo* through pharmacodynamic assessment of the ocular pressure reduction, drug concentration-time profiles in ocular tissues were not determined. Nevertheless, the assessed pharmacodynamic response reflects direct evidence of the pharmacological activity and therapeutic behavior of the CTC/NFD-OF gel. Incorporating pharmacokinetic analyses would offer more insights into the correlation between ocular drug disposition and the observed pharmacodynamic response. Additionally, although gamma irradiation was employed as a terminal sterilization step, validated sterility testing was not applied to check and verify the sterility assurance of CTC/NFD-OF gel. Therefore, further studies should consider formal sterility testing and validation of the sterilization process to confirm the microbiological quality and sterility of the formulation. Another limitation of the study is the absence of a blank hyalurosomal gel and single drug groups in the pharmacodynamic study, as the blank hyalurosomal gel and single drug assessment are essential to distinguish carrier effects from the benefits of combined therapy. We also acknowledge that further evaluation in an appropriate experimentally induced glaucoma model would be necessary to confirm therapeutic efficacy under disease conditions.

## 5 Conclusion

The CTC/NFD-OF gel serves as a multifunctional platform, combining the integral inherent features of its components. It can be considered an innovative therapy for the topical control of glaucoma through multiple cooperative and interwoven mechanisms. In this study, NFD was successfully loaded into hyalurosomal gel, to which CTC was subsequently added, aiming to emphasize the importance of combined therapy in an appropriate nano delivery system, which can bypass the main vulnerabilities of both the drug and the administration route. Many *in-vitro* and *in-vivo* evaluations were conducted in a sequential paradigm to ensure the safety and efficacy of the CTC/NFD-OF gel. NFD-OF loaded in the gel showed nanosized vesicles with an appropriate EE of 70.7% ± 2.3%, ZP of −22.8 ± 1.3 mV, and sustained % NFD and CTC released (65.5% ± 1.3% and 99.5% ± 0.8%, respectively) up to 24 h, and the CTC/NFD-OF gel was successfully sterilized by gamma radiation and showed a powerful corneal permeation in addition to tolerability by ocular tissues. Moreover, a pronounced enhancement in pharmacodynamic parameters was observed after topical administration of CTC/NFD-OF gel. Herein, it can be concluded that the CTC/NFD-OF gel offers a substantial advance in the evolution of combined therapy for glaucoma, supporting patients’ compliance and welfare.

Future studies are recommended to further investigate the therapeutic potential of the developed CTC/NFD-OF gel in diseased glaucoma animal models, as well as to evaluate its cellular antioxidant and relevant neuroprotective effects. In addition, comprehensive clinical studies are needed to confirm the long-term ocular tolerability, therapeutic efficacy, and patient compliance of the developed CTC/NFD-OF gel as a promising anti-glaucoma therapy.

## Data Availability

The original contributions presented in the study are included in the article/supplementary material, further inquiries can be directed to the corresponding authors.
